# The cooling tower water microbiota: Seasonal dynamics and co-occurrence of bacterial and protist phylotypes

**DOI:** 10.1016/j.watres.2019.04.028

**Published:** 2019-08-01

**Authors:** Han-Fei Tsao, Ute Scheikl, Craig Herbold, Alexander Indra, Julia Walochnik, Matthias Horn

**Affiliations:** a Centre for Microbiology and Environmental Systems Science, University of Vienna, Vienna, Austria; b Institute of Specific Prophylaxis and Tropical Medicine, Medical University of Vienna, Vienna, Austria; c Department of Mycobacteriology and Clinical Molecular Biology, AGES, Vienna, Austria

**Keywords:** Microbial diversity, Legionella, Mycobacteria, Chlamydia, Protists, Free-living amoeba, Built environment, HVAC cooling tower

## Abstract

Cooling towers for heating, ventilation and air conditioning are ubiquitous in the built environment. Often located on rooftops, their semi-open water basins provide a suitable environment for microbial growth. They are recognized as a potential source of bacterial pathogens and have been associated with disease outbreaks such as Legionnaires’ disease. While measures to minimize public health risks are in place, the general microbial and protist community structure and dynamics in these systems remain largely elusive. In this study, we analysed the microbiome of the bulk water from the basins of three cooling towers by 16S and 18S rRNA gene amplicon sequencing over the course of one year. Bacterial diversity in all three towers was broadly comparable to other freshwater systems, yet less diverse than natural environments; the most abundant taxa are also frequently found in freshwater or drinking water. While each cooling tower had a pronounced site-specific microbial community, taxa shared among all locations mainly included groups generally associated with biofilm formation. We also detected several groups related to known opportunistic pathogens, such as *Legionella*, *Mycobacterium*, and *Pseudomonas* species, albeit at generally low abundance. Although cooling towers represent a rather stable environment, microbial community composition was highly dynamic and subject to seasonal change. Protists are important members of the cooling tower water microbiome and known reservoirs for bacterial pathogens. Co-occurrence analysis of bacteria and protist taxa successfully captured known interactions between amoeba-associated bacteria and their hosts, and predicted a large number of additional relationships involving ciliates and other protists. Together, this study provides an unbiased and comprehensive overview of microbial diversity of cooling tower water basins, establishing a framework for investigating and assessing public health risks associated with these man-made freshwater environments.

## Highlights

• Highly dynamic bacterial and protist community structure in cooling towers.
• Cooling tower water basin communities resemble other open freshwater environments.
• Core bacterial microbiome of cooling towers consists of biofilm-forming taxa.
• Co-occurrence network analysis suggests importance of inter-kingdom relationships.

## Graphical abstract

**Figure:**
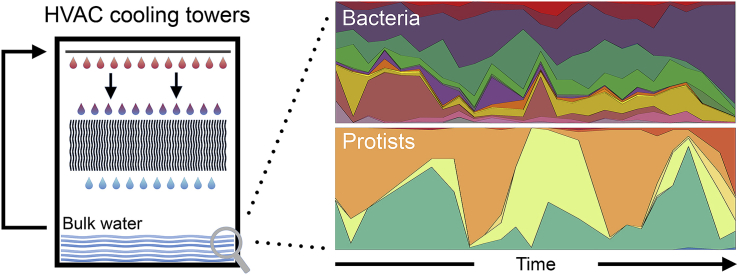

## 1 Introduction

Most modern commercial, industrial, and residential buildings rely on cooling towers as cost-efficient measures to remove excess heat. Containing large semi-open water volumes at a rather constant temperature, cooling towers are suitable environments for microbial growth throughout the year and have been implicated in bacterial outbreaks (Kurtz et al., 1982; Pagnier et al., 2009; Torvinen et al., 2013; Yamamoto et al., 1992). One of the major public health risks associated with cooling towers is Legionnaires’ disease. This respiratory tract infection is caused by *Legionella pneumophila*, a gammaproteobacterial pathogen acquired through inhalation of aerosols and is potentially fatal for immunocompromised patients (Hamilton et al., 2018; Walser et al., 2014). Other opportunistic bacterial pathogens detected in cooling towers include *Mycobacterium* spp., *Pseudomonas* spp., *Burkholderia* spp., and *Pantoea* spp. (Ceyhan and Ozdemir, 2008).

Protists, such as free-living amoebae, are common members of microbial communities in cooling towers (Barbaree et al., 1986; Berk et al., 2006; Delafont et al., 2016; Pagnier et al., 2009). They are predators of other microbes in the system and may also be reservoirs for bacterial pathogens such as *Legionella* and *Mycobacterium* spp., *Pseudomonas aeruginosa*, *Francisella tularensis*, *Coxiella burnetii,* and *Vibrio cholerae* (Abd et al., 2003; La Scola and Raoult, 2001; Sandström et al., 2010; Thomas and McDonnell, 2007; Thom et al., 1992; Van der Henst et al., 2016). These microbes are able to escape the regular phagolysosomal pathway and transiently replicate within amoeba trophozoites. Moreover, many free-living amoebae can form cysts, a resistant life stage providing protection from unfavorable environmental conditions including biocides (Fouque et al., 2012; Miltner and Bermudez, 2000). Efficient disinfection procedures targeting not only bacteria but also protists have thus been recognized as important in reducing the public health risk associated with cooling towers (Critchley and Bentham, 2009; Scheikl et al., 2016).

Bacteria-protist relationships are manifold (Cirillo et al., 1997, 1994; Fritsche et al., 1998; Greub and Raoult, 2004; Hess, 2016; Hess, 2017; Molmeret et al., 2005). They also include obligate bacterial symbionts, such as *Caedibacter* and *Holospora* species in ciliates (Dziallas, 2012; Fokin, 2004; Goertz, 2001), or *Protochlamydia* and *Parachlamydia* species, also known as environmental chlamydiae (Horn, 2008; Taylor-Brown et al., 2015). Yet, microbial communities and their interactions in cooling tower water are likely far more complex than recognized. The presence of specific bacterial pathogens has been investigated (Inoue et al., 2015; Li et al., 2015; Liu et al., 2011, 2009; States et al., 1987; Torvinen et al., 2013; Ulleryd et al., 2012), but surveys on the overall microbial community composition are rare (Llewellyn et al., 2017; Wang et al., 2013). Studies specifically investigating community assembly and dynamics in these systems are largely lacking, with currently only one recent report (Pereira et al., 2017). Except for a few notable studies (Llewellyn et al., 2017; Pereira et al., 2017; Wang et al., 2013), work on the microbial community of water towers has been based on cultivation dependent methods or PCR assays targeting specific microbial groups, which are inherently limited in both taxonomic resolution and scope. High-throughput DNA sequencing of 16S/18S ribosomal RNA gene PCR products, termed amplicon sequencing, is able to circumvent these limitations and provides detailed data on both bacterial and protist communities (Thompson et al., 2017). After all, it is the wide range of interactions - from antagonistic to synergistic - that shape microbial assemblages and ultimately determine the chances for pathogen proliferation.

In this study, we used amplicon sequencing to investigate seasonal and geographic variation in the composition of bacterial and protist communities found in three cooling towers over a one-year period. We focused our analysis on the bulk water in the water basins, because it is the source of aerosols formed during cooling tower operation (Nhu Nguyen et al., 2005). Bacteria-protist relationships were inferred from co-occurrence network analysis. These approaches identified key microbial players and provided detailed insight into the microbial community dynamics in these systems including putative bacteria-protist interactions.

## 2 Materials and methods

### 2.1 Site description, sample collection, and processing

Three different heating, ventilation and air conditioning (HVAC) cooling towers located on the rooftop of different buildings in the city of Vienna, Austria, were studied. Two buildings, a hospital and a business complex, were located near the city center (1.7 km apart from each other) whereas a second hospital was located at Vienna's periphery (5 km from the other two locations). The two hospitals are hereafter referred to as (“cooling tower 1”) CT-1 and CT-2, the business complex as CT-3. For all three sites, the water temperature was recorded throughout the sampling campaign. The cooling towers were subjected to disinfection with chlorine/bromine-based disinfectant (1–3 g/m^3^) over an automated dosimeter. CT-1 was disinfected three times a week, CT-2 every 18 h and CT-3 every four days.

During the sampling period between September 2013 and September 2014, water samples were taken on a biweekly basis. CT-3 was not operating during the winter season, resulting in fewer samples. From the tank basin, 3 L of bulk water was sampled and stored at 4 °C for up to a day before further processing. The water was stirred first and then 2 L per sample were vacuum-filtered onto a cellulose nitrate filter (diameter 0.2 μm; 12.5 cm^2^; Sartorius stedim) in 500 ml steps. In case of rapid filter clogging, filters with a slightly larger pore size (diameter 0.45 μm) were used in addition for further filtration of the same sample. Up to four filters were used per sample. All filters were subsequently used for downstream processing.

### 2.2 DNA extraction, and 16S/18S rRNA gene amplicon sequencing

The PowerWater^®^ DNA Isolation Kit (Qiagen, Hilden, Germany) was used for DNA extraction from the filters. All steps were performed according to the standard centrifuge-based protocol recommended by the manufacturer. Briefly, the filters were inserted in a lysis buffer-containing bead beating tube where the cells were mechanically and chemically lysed. The lysates were transferred to a DNA-retaining spin column; lysates from the same sample were loaded onto the same column to pool the DNA. After washing, DNA was eluted in the DNA elution buffer and used for PCR.

A two-step barcoded amplicon sequencing approach was used as described previously (Herbold et al., 2015). Briefly, fragments of the bacterial and eukaryotic small subunit rRNA gene were amplified, resulting in 26 libraries each for CT-1 and CT-2, and 14 libraries for CT-3. V3 and V4 regions of the bacterial 16S rRNA gene were amplified with the primers Bakt_341F (5′-CCTACGGGNGGCWGCAG-3′) and Bakt_805R (5′-GACTACHVGGGTATCTAATCC-3’; Herlemann et al., 2011). Archaea were not targeted by this primer set and were not analysed in this study. The eukaryotic 18S rRNA gene primer-pair EUK_1391F (5′-GTACACACCGCCCGTC-3′) and EUK_1510R (5′-CCTTCYGCAGGTTCACCTAC-3′) based on the Earth Microbiome Project (Version 5, 2012; Amaral-Zettler et al., 2009; Gilbert et al., 2014) were used to amplify the 18S—V9 region. For each primer set and sequencing run, negative controls without the addition of DNA were performed and sequenced.

Each PCR reaction included 1x DreamTaq Green Buffer (Fermentas, Thermo Fisher Scientific, Vienna, Austria), 2 mM MgCl_2_, 0.2 mM dNTP mix (Fermentas), 0.1 mg mL^−1^ bovine serum albumin, 1 μM of each of the forward and reverse primers, 0.025 U DreamTaq polymerase (Fermentas), and 1 μL of DNA template. This first PCR amplification (94 °C for 3 min; 25 cycles of 45 s at 94 °C, 30 s at 52 °C (16S rRNA gene) or 57 °C (18S rRNA gene), 90 s at 72 °C; and 72 °C for 10 min) was performed in triplicates; PCR products were pooled and served as the template for the second barcoding PCR (95 °C for 3 min; 10 cycles of 30 s at 95 °C, 30 s at 55 °C, 60 s at 72 °C; and 72 °C for 7 min). Purification and quantification of PCR products were done as described (Herbold et al., 2015). The TruSeq Nano DNA Library Prep Kit (Illumina) was used for adaptor ligation and PCR without the fragmentation step. Sequencing was performed by Microsynth AG (Balgach, Switzerland) on a MiSeq system (Illumina) using the MiSeq Reagent kit V3. Resulting sequence datasets were deposited in the NCBI Sequence Read Archive under study accession number PRJEB21563.

### 2.3 Data compilation, filtering, clustering, and classification

Paired-end reads were demultiplexed and trimmed as described (Herbold et al., 2015) and were assembled using fastq-join within Qiime (Caporaso et al., 2010). 18S rRNA reads were forced to be trimmed by at least 88 nucleotides prior to trimming and assembly because the expected amplicon length of 178 bp was shorter than the average read length. Clustering into operational taxonomic units (OTUs) and chimera-checking were performed using Uparse (Edgar, 2013), using a distance of 0.03 and disallowing singletons to serve as a centroid. Taxonomic classification of OTU centroids was carried out using the Bayesian classifier implemented in mothur (Schloss et al., 2009) and the Silva SSU database (Version 132; Quast et al., 2013) for 16S rRNA data sets, or the protist ribosomal reference database PR^2^ (Version 4.11.1; Guillou et al., 2013) for 18S rRNA data sets. OTUs with a classification confidence score lower than 80% for 16S rRNA data sets and 60% for 18S rRNA data sets were changed to ‘unclassified’. The class Betaproteobacteria has recently been proposed to be reclassified as the new order Betaproteobacteriales within the class Gammaproteobacteria (Parks et al., 2018). For clarity and consistency with previous studies, the Betaproteobacteria were still treated as a separate class, but both names are provided. 16S rRNA OTUs classified as mitochondria and chloroplasts were omitted from subsequent analysis; this did not change the overall estimates of alpha and beta diversity measures. 16S rRNA OTUs present only in the negative controls or strongly overrepresented (50 fold) in the negative controls compared to all samples were considered to be cross-contamination and thus removed from the dataset (Table S1).

All 18S rRNA OTUs only present in the negative control were removed from the dataset. OTUs present in both control and samples were only retained if their sample-abundance exceed the control-abundance by 1000 fold (Table S2). Taking into account the known relationships between protists and pathogenic bacteria in the water-borne environment, we restricted our analysis of 18S rRNA sequences to the groups Amoebozoa (Lobosa, Conosa), Excavata (Discoba, Metamonada), Rhizaria (Cercozoa, Foraminifera) and Alveolata (Apicomplexa, Ciliophora, Dinoflagellata). Both data sets were rarefied individually to standardize sequence numbers using the rarify_even_depth function in the R-package phyloseq (McMurdie and Holmes, 2013) with replacement option turned off. For the 16S rRNA data set a minimum of 1713 sequences was used, representing the 15% quantile of the data; for the 18S rRNA data set, which had a greater library size variation, a cutoff of 59 sequences equivalent to 45% quantile of the data was used. Samples not fulfilling these criteria were removed from the subsequent analysis.

### 2.4 Alpha and beta diversity measures and co-occurrence analysis

Alpha diversity was assessed for both data sets with the R-package *phyloseq* (McMurdie and Holmes, 2013) using the estimators Chao1 and the Shannon diversity index. Bacterial community composition was compared by non-metric multidimensional scaling (NMDS) analysis based on Bray-Curtis dissimilarity.

For the OTU co-occurrence analysis, only those samples for which both 16S and 18S rRNA sequence data were available after rarefying were included. In addition, only OTUs present in at least 3 samples (for each cooling tower) and with a total number of sequences greater than 10 were kept to remove the number of infrequent OTUs. For each cooling tower, co-occurrence analysis of 16S rRNA and 18S rRNA OTUs was performed using CoNet (Faust and Raes, 2016), a plug-in of the software Cytoscape (Shannon et al., 2003). To calculate consensus networks, a combination of different correlation and distance measures including Pearson and Spearman correlation, mutual information, Kullback-Leibler divergence, and Bray-Curtis dissimilarity was used. The maximum lag was set to 1 to include slightly shifted associations. Distribution of all pair-wise scores was generated, and the 7.5% top-scoring edges supported by at least four out of five (positive correlations) or three out of five (negative correlations) measures were kept. This process was performed with 1000 renormalized permutations, resampling by row-shuffling, and p-value merging with Brown's method. After applying Benjamini-Hochberg's false discovery rate correction, edges with a corrected p-value below 0.05 were kept.

The R-package ampvis (Albertsen et al., 2015) was used to generate heatmaps; MetacodeR (Foster et al., 2017) was used to generate heat trees.

## 3 Results and discussion

In this study, we monitored the microbial community in the water basins of three heating, ventilation and air conditioning (HVAC) cooling towers - in the following referred to as CT-1, CT-2, and CT-3 - over the course of one year. The temperature in the basins ranged between 20.1 °C and 32.3 °C for CT-1 (99 percentile of all temperature data points), and between 19.9 °C and 29 °C for CT-2 throughout the sampling period. The average temperature during the summer months was slightly higher than during winter; 27.8 °C vs. 26.4 °C for CT-1, and 26.6 °C vs. 23.1 °C for CT-2. Temperature data for CT-3 were only available after mid-April 2014; its temperature range was 10.6–27.4 °C, larger than that of the other two towers. Elevated temperature in the water basin is a characteristic feature of cooling towers and together with the semi-open design of these systems provide good conditions for microbial growth. Cooling tower water tanks, like other freshwater or drinking water systems, develop biofilms (Liu et al., 2009). Yet, for the purpose of this study we focused on the analysis of bulk water for two reasons: (i) bulk water represents the seed community for biofilm formation in the basin, and (ii) it is the source of aerosols formed during cooling tower operation (Nhu Nguyen et al., 2005).

### 3.1 Complex but reduced bacterial diversity compared to open freshwater environments

In total, we collected 26 samples each for CT-1 and CT-2, and 14 samples for CT-3. Amplicon sequencing of 16S and 18S rRNA genes revealed taxonomically diverse bacterial and protist communities for all three cooling towers. To study seasonal dynamics of microbial diversity in the three cooling towers, we first estimated species-level richness and evenness using a threshold of 97% sequence similarity for operational taxonomic unit (OTU) definition. Good's coverage estimator was 0.984 ± 0.007 (mean ± SEM) for 16S OTUs and 0.958 ± 0.018 (mean ± SEM) for 18S OTUs, indicating that sequencing depth was sufficient for reliable community composition analysis. After rarefying, our data set included 23 CT-1 samples, 21 CT-2 samples, and 12 CT-3 samples. In total 590 bacterial OTUs and 321 protist OTUs were considered for our analysis (Tables S3 and S4).

**Fig. 1:**
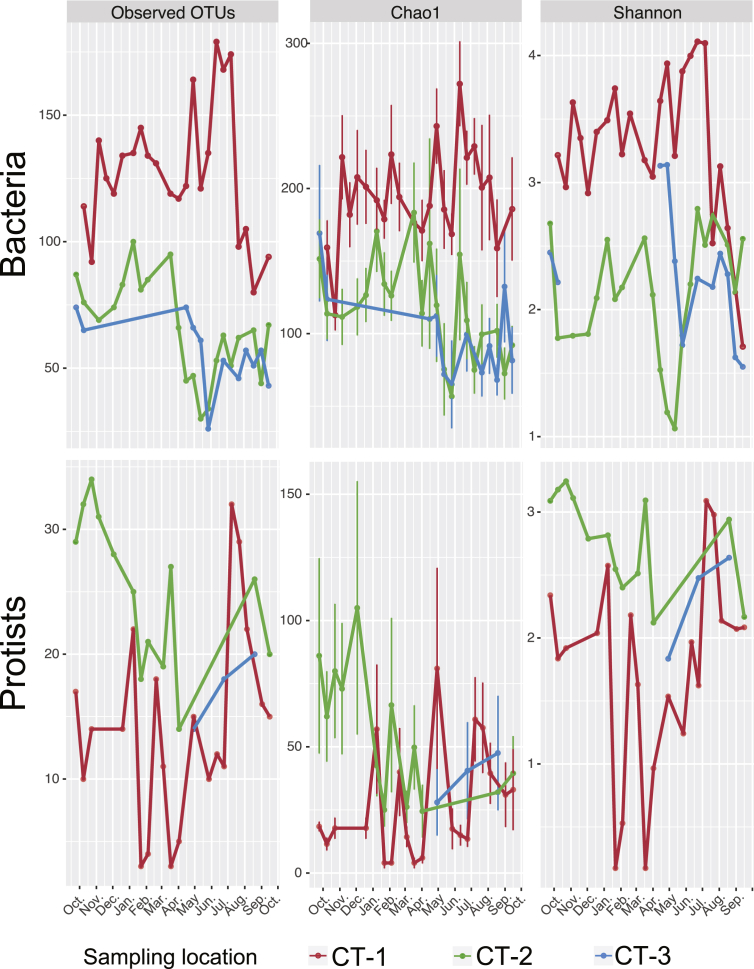
**Bacterial and protist diversity in three cooling tower water basins.** The number of observed OTUs and diversity indices for estimation of species richness and evenness of bacterial and protist communities are indicated for cooling towers CT-1, CT-2, and CT-3 over a sampling period of one year (September 2013 to September 2014). Pronounced differences between sampling sites and time points in both bacterial and protist diversity can be observed.

**Fig. 2:**
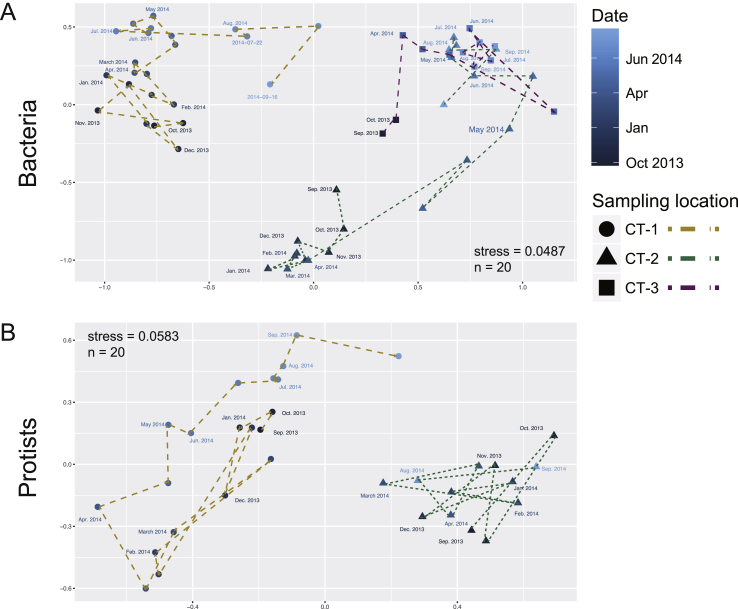
**Similarity of bacterial and protist community structure across time and space.** Nonmetric multidimensional scaling (NMDS) analysis illustrating beta diversity among all samples based on the Bacterial or protist community composition (protist diversity was not included in CT-3 samples due to low sample number). For each cooling tower, the samples were sequentially connected with arrows. The first sample of each month are annotated with the date in the plot. Microbial communities of cooling tower CT-1 are clearly separated in this analysis, indicating a unique community composition. Shifts in bacterial community structure apparently follow a seasonal trend.

The three cooling towers showed clear differences in bacterial and protist species richness and evenness (Fig. 1). As a general pattern, protist diversity was much lower than bacterial diversity for all samples and sampling sites. Microbial diversity fluctuated strongly throughout the sampling period, and there was no clear common trend observed among the different cooling towers. It is interesting that while CT-1 had the highest bacterial diversity values (as inferred from Chao1 and Shannon estimators; Welch's *t*-test p-value < 0.0001), it also showed the lowest protist diversity and evenness compared to the other two locations, which were also more similar with each other (Fig. 1). This was also reflected in the NMDS-based beta-diversity analysis (Welch-corrected *t*-test of the average Bray-Curtis distance within one sampling site compared to other sites; p-value < 0.0001), revealing clear sample clusters according to sampling site and time point (Fig. 2). At all sampling sites, we observed gradual community shifts over time reflecting seasonal changes in bacterial and protist diversity. In CT-1, the high similarity between autumn, winter, and spring samples indicated a gradual rather than sudden change. In contrast, the community shift in CT-2 was more pronounced (Fig. 2).

**Fig. 3:**
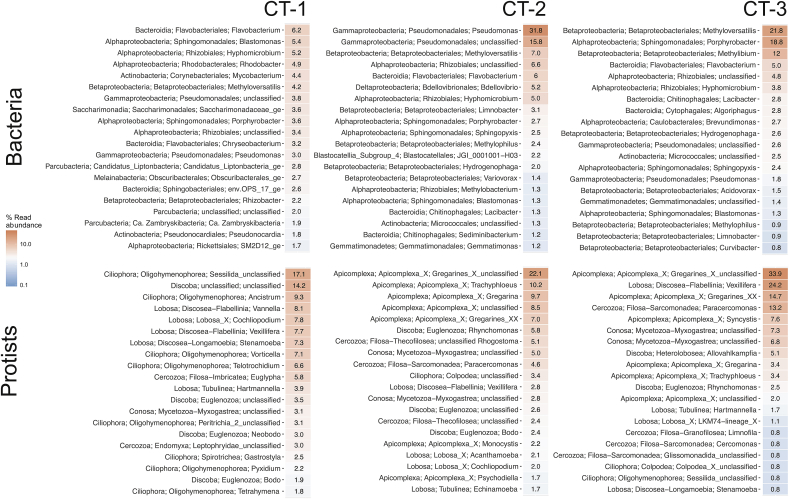
**The most abundant bacterial and protist genus at three cooling towers.** For each sampling site, the average relative abundance of the 20 most frequently observed bacteria and protist taxa are listed at the genus level. In total 79% of all bacterial OTUs and 59% of all protist OTUs could be classified at the genus level (Tables S3 and S4). For clarity, the phylum as well as the order level are also indicated (the Proteobacteria were split into its representative classes). The Sphingomonadales, Pseudomonadales, Rhizobiales, and Burkholderiales including many biofilm associated taxa are among the top bacterial groups.

To better understand community structure we next classified all OTUs, grouped them at the genus level, and focused on the 20 most abundant genera (Fig. 3). CT-2 and CT-3 are both dominated by a few abundant genera, whereas in CT-1, abundances of the top genera are more evenly distributed. This analysis showed that the general bacterial community composition is different between the three cooling towers, yet *Flavobacterium* (Bacteroidetes), *Hyphomicrobium,* members of the Rhizobiales and Sphingomonadales (Alphaproteobacteria), *Pseudomonas* (Gammaproteobacteria), and *Methyloversatilis* (Betaproteobacteria/Betaproteobacteriales; Parks et al. 2018) are among the top abundant taxa in all three towers (Fig. 3). The most striking difference between the cooling towers is the extremely high *Pseudomonas* abundance in CT-2 (Fig. 3), which makes up almost 31.8% of all sequence reads (compared to up to 3% at the other two sampling sites) while other Gammaproteobacteria were generally not well represented in our data set. Our observations are consistent with findings from the only cooling tower for which a similar analysis is available so far (Pereira et al., 2017). In this study, members of the Rhizobiales, Burkholderiales, Methylophilales, and Cytophagales were also among the most abundant taxa. In contrast, we didn't detect Xanthomonadales among the top taxa, but found other groups to be well represented, including Corynebacteriales, Rhodocyclales, and Pseudomonadales (Fig. 3). It is noteworthy, that Betaproteobacteria (Betaproteobacteriales), commonly found in freshwater systems (Newton et al., 2011), are with two exceptions absent in the CT-1 top genera list, whereas they are abundant in CT-2 and CT-3.

Two members of the recently identified Candidate Phyla Radiation (CPR) are among the most abundant taxa in CT-1: Parcubacteria (formerly OD1) and Saccharimonadia (formerly known as TM7), both now classified within the phylum Patescibacteria. Members of both groups show reduced genomes and only limited metabolic capabilities, likely endorsing a parasitic or symbiotic lifestyle (He et al., 2014; Nelson and Stegen, 2015). They are known to have small cell sizes and are able to pass through a 0.22 μm filter (Luef et al., 2015). CT-1 also included an unclassified member of the Rickettsiales (Alphaproteobacteria) among the top abundant taxa, again a group of microbes generally associated with an intracellular lifestyle.

Overall, the most abundant bacterial orders identified in the cooling tower samples are well in agreement with other reports on man-made water distribution systems, where orders belonging to Alpha-, Beta-, and Gammaproteobacteria are usually the prevalent taxa (Berry et al., 2006; Brown et al., 2015; Farenhorst et al., 2017; Kalmbach et al., 1997; Llewellyn et al., 2017; Martiny et al., 2005; McCoy and VanBriesen, 2012; Pereira et al., 2017; Revetta et al., 2010; Tokajian et al., 2005; Vaz-Moreira et al., 2012; Wang et al., 2013; Zhang et al., 2012). Bacterial communities in more natural settings such as lakes, rivers, or streams are often dominated by Betaproteobacteria (Betaproteobacteriales), but also include Actinobacteria, Bacteroidetes, Cyanobacteria, Verrucomicrobia, and Saccharimonadia (Boucher et al., 2006; Crump and Hobbie, 2005; Liu et al., 2012; Lozupone and Knight, 2005; Mueller-Spitz et al., 2009; Newton et al., 2011; Tang et al., 2015; Zwart et al., 2002). The dominance of these groups in the cooling tower microbial community structure is consistent with what has been reported for other freshwater systems. This suggests that community structure in cooling towers may be primarily influenced by the seed community in the incoming drinking water and then shaped by the specific conditions in the cooling tower water basin.

### 3.2 Seasonal microbial community dynamics

**Fig. 4:**
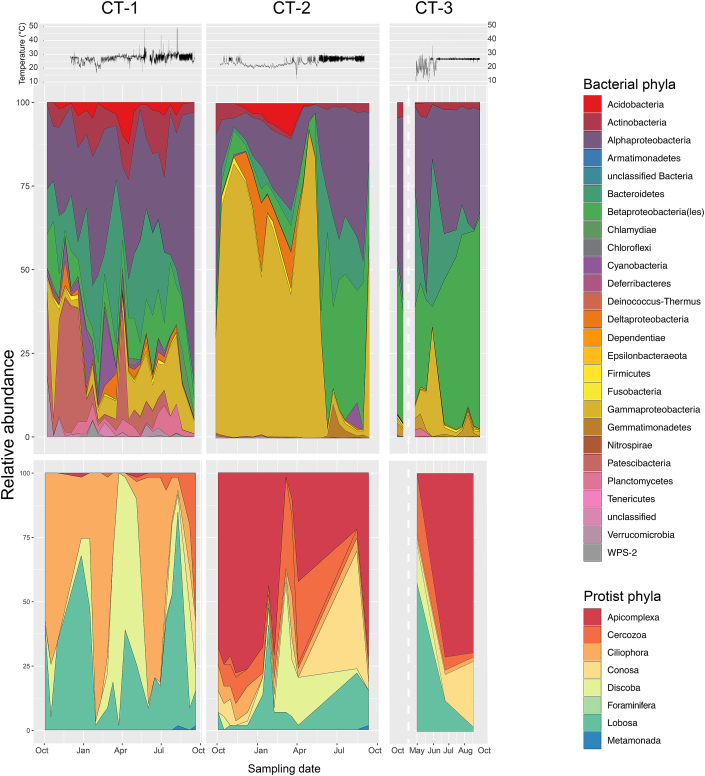
**Temporal dynamics of bacterial and protists diversity over a one year sampling period.** Changes in relative abundance of bacteria and protists at the phylum level for cooling towers CT-1, CT-2, and CT-3 reveals temporal dynamics of community structure despite relatively constant environmental conditions. The average daily temperature data in the water basin is shown in the uppermost panel; lack of data for CT-3 is indicated by a white dashed line.

To further analyse temporal changes in community structure, we examined the relative abundance of phyla over time. Consistent with our previous analysis, we observed pronounced changes in bacterial community composition over time (Fig. 4). The most dramatic seasonal change occurred in cooling tower CT-2, where an abrupt decline of Gammaproteobacteria (*Pseudomonas*) was observed during June 2014 (Fig. 4). Pronounced variations were also observed in the more diverse community of CT-1. In CT-3, the overall bacterial community composition was rather stable compared to the other cooling towers (Fig. 4). Protistan community members of the Rhizaria, the Excavata, the Alveolata, and the Amoebozoa were found at all three sampling sites, with the latter two phyla being most abundant and showing pronounced temporal variations. Notably, changes in bacterial and protist diversity do not necessarily follow the same trend, indicating complex interactions and differences in response to environmental conditions (Fig. 3, Fig. 4).

Next, we aimed to understand differences and similarities between bacterial and protist community composition and dynamics in the three cooling towers in more detail. To this end, we focused on various sets of OTUs: We defined a set of persistent OTUs as taxa that were detected at a given location at nearly all time points (taxa were allowed to be missing from only two samples, corresponding to at least 90% of all samples for CT-1 and CT-2, and 80% for CT-3). The second set was referred to as site-specific OTUs and included those taxa that were exclusively found at a single location. The third set comprised taxa detected at all cooling towers and was referred to as shared OTUs.

**Fig. 5:**
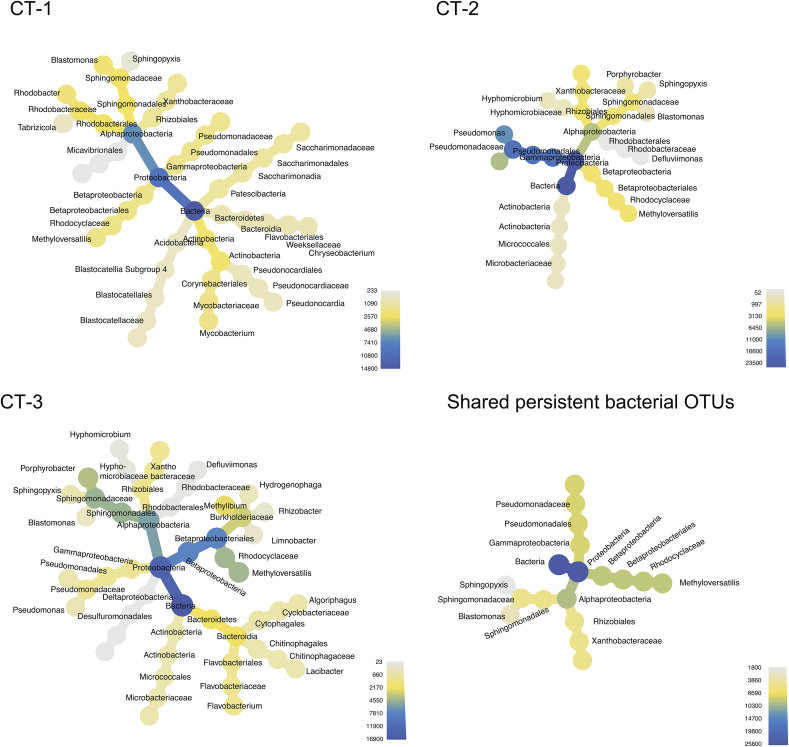
**Persistent bacterial OTUs in three cooling tower water basins.** For each sampling site, a heat tree depicts the persistent bacterial taxa at the genus level (unclassified taxa are unlabeled). Persistent taxa are defined as being present in nearly all samples of one sampling location. The color corresponds to the total number of sequences detected for each sampling site at the respective taxonomic level. Shared bacterial OTUs are persistent taxa found in all three sampling sites, all belonging to the Proteobacteria. (For interpretation of the references to color in this figure legend, the reader is referred to the Web version of this article.)

Consistent with our observed seasonal changes, there were few persistent OTUs, 13 in CT-1, 10 in CT-2, and 18 in CT-3 (Fig. 5). They included Actinobacteria (*Mycobacterium* sp., or members of the Microbacteriaceae), Proteobacteria (*Sphingopyxis, Methyloversatilis, Blastomonas, Defluviimonas*, *Porphyrobacter,* Xanthobacteraceae), Patescibacteria (Saccharimonadia, formerly TM7; Albertsen et al., 2013), Bacteroidetes, or Acidobacteria. Interestingly, most of the top five abundant taxa in each of the cooling towers also belonged to the set of persistent OTUs (Fig. 3, Fig. 5).

**Fig. 6:**
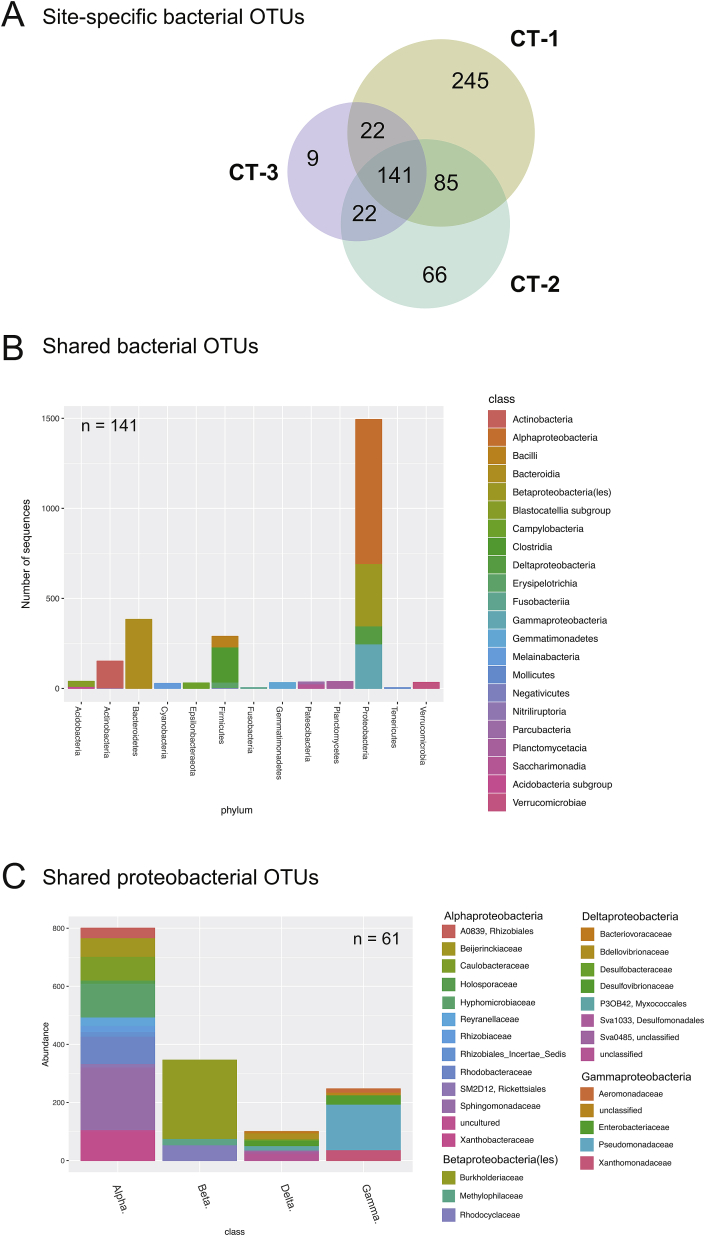
**Site-specific and shared bacterial OTUs in three cooling towers. (A)** The Venn diagram represents the proportion of shared and site-specific bacterial OTUs at the three sampling sites. Reflecting its higher species richness, CT-1 showed the greatest number of site-specific bacterial OTUs. The number of OTUs shared by all three sampling sites clearly exceeds the number of OTUs shared individually by two cooling towers. **(B)** The bar chart shows the taxonomic classification of the shared OTUs (occurring at least once at each sampling site). The majority of the OTUs belongs to the Proteobacteria, Bacteroidetes, Actinobacteria, and Firmicutes. **(C)** A more detailed view of the shared Proteobacteria on the family level. The most abundant groups are the Bradyrhizobiaceae, Caulobacteraceae, Hyphomicrobiaceae, Sphingomonadaceae, Comamonadaceae, and Pseudomonadaceae.

The differences between the cooling towers were further analysed by focusing on the set of site-specific taxa. Consistent with earlier observations (Fig. 1), CT-1 had by far the largest number of site-specific bacterial OTUs (245, 53.1.%), compared to CT-2 (66, 14.3%) and CT-3 (9, 1.9%; Fig. 6A). It is noteworthy, that there is no overlap between the sets of site-specific taxa and the persistent OTUs for any of the cooling towers. In direct comparison, the site-specific taxa comprised a wider range of diverse phyla, including members of the Patescibacteria, Chlamydiae, Chloroflexi, Planctomycetes, Verrucomicrobia, and Depedentiae (TM6; Delafont et al., 2015; Table S3). The transient character of the site-specific OTUs was further illustrated by most of them being detected at a maximum of nine time points. This indicated that there was a substantial fraction of taxa specific to individual cooling towers that failed to establish long-term colonization.

### 3.3 The core bacterial microbiome of cooling towers consists of biofilm-forming taxa

To better understand the common features of cooling tower microbial communities, we next focused on the set of shared OTUs, i.e. those detected at least once at more than one site. This set represents 30.6% of all bacterial OTUs in this study, and the fraction of shared OTUs varied between the individual cooling towers (28.6%, 44.9%, and 72.6%, respectively). The majority of shared OTUs belongs to the Alphaproteobacteria (Sphingomonadaceae and Hyphomicrobiaceae), Betaproteobacteria/Betaproteobacteriales (Burkholderiaceae), Actinobacteria, Bacteroidetes (Bacteroidia), and Gammaproteobacteria (Pseudomonadaceae) (Fig. 6B and C). From this large set of shared OTUs only six bacterial taxa were nearly always present, i. e. also belong to the set of persistent OTUs (Fig. 5). This included members of the genera *Pseudomonas, Methyloversatilis*, *Blastomonas, Sphingopyxis,* and *Porphyrobacter*, and the family Xanthobacteraceae (formerly classified as Bradyrhizobiaceae).

A common feature of these microbes is their ability to form or to be associated with microbial biofilms (Rickard et al., 2004; Soto-Giron et al., 2016; Zhang et al., 2012). *Pseudomonas* species are ubiquitous environmental bacteria well-known for their capability to form biofilms (Costerton, 1999; Jensen et al., 2010; Sauer et al., 2002). They are considered pioneers, specifically facilitating the initiation of biofilm formation (Douterelo et al., 2014). Members of the genus *Methyloversatilis* are found in various environments including drinking water and biofilms (Kalyuzhnaya et al., 2006; Liu et al., 2012; Shpigel et al., 2015; van der Kooij et al., 2017; Williams et al., 2004). *Blastomonas* species have the ability to coaggregate with various bacteria, an important feature for biofilm formation (Katharios-Lanwermeyer et al., 2014; Rickard et al., 2002). Additionally, other members of the order Sphingomonadales such as *Sphingopyxis*, *Porphyrobacter*, and the family Xanthobacteraceae (formerly Bradyrhizobiaceae) were repeatedly found to be associated with bacterial biofilms and are commonly present in drinking water environments (Bereschenko et al., 2010; Buse et al., 2014; Chao et al., 2015; Douterelo et al., 2014; Hong et al., 2010; Muchesa et al., 2017; Revetta et al., 2010; Rożej et al., 2015; Singh et al., 2003; Vaz-Moreira et al., 2011).

The occurrence and persistence of these microbes at all cooling towers suggest that biofilm formation is an important process in cooling tower microbial communities, likely contributing to maintenance and resistance against disinfection measures. Cooling tower basins offer a large surface area and are characterized by a slow water flow speed, a rather constant and elevated temperature, as well as increased concentrations of organic matter compared to closed drinking water systems. These conditions are well-suited for biofilm formation (Türetgen and Cotuk, 2007; Zacheus et al., 2000).

Biofilms are preferred niches of protists because of the high density of microbes serving as a potential food source, and grazing is a major factor modulating the structure and function of bacterial biofilms (Flemming et al., 2016; Matz et al., 2005; Parry, 2004). Biofilms are also a known reservoir and potential source for a number of bacterial pathogens (Delafont et al., 2014; Garcia et al., 2013; Greub and Raoult, 2004; Wingender and Flemming, 2011).

### 3.4 Opportunistic bacterial pathogens

Over the course of our one-year sampling campaign we detected few OTUs classified as genera comprising known (opportunistic) bacterial pathogens, as defined in a previous study (Pereira et al., 2017; Table S5). The abundance of these OTUs ranges from 0.01% to 28% depending on the sampling location, but the majority are below 1%. We observed a total of 29 OTUs belonging to three different phyla, Actinobacteria, Firmicutes, and Proteobacteria. This includes *Bosea*, *Helicobacter,* and *Stenotrophobacter* and several *Arcobacter*, *Bacillus*, *Sphingomonas, Legionella*, *Mycobacterium,* and *Pseudomonas* species (Table S5). In comparison to the cooling tower studied earlier, the cooling towers investigated here display a less diverse set of genera including potential pathogens (Pereira et al., 2017).

Of note, none of the *Legionella* sequences showed a particularly high similarity to the human pathogen *Legionella pneumophila*. This is consistent with no *L. pneumophila* outbreak having been reported in the vicinity of our sampling locations during this study. The *Legionella* species detected belong to a group commonly referred to as *Legionella*-like amoebal pathogens, a large number of species primarily found as parasites in free-living amoebae (Greub and Raoult, 2004; Marrie et al., 2001; Newsome et al., 1988). All *Legionella* OTUs belong to the set of site-specific taxa, and their relative abundances were below 0.1%, rendering them members of the rare biosphere in these systems.

We found four OTUs assigned to the genus *Mycobacterium*, two of which occurred in two cooling towers. One of those, *M. wolinsky*, showed a relatively high abundance in CT-1 (4%) and belonged to the persistent taxa in this cooling tower (Table S3, Fig. 5). *M. wolinsky* can cause bacteremia and, like the other mycobacteria detected in this study, is a member of the nontuberculous mycobacteria (NTM) group. This group includes opportunistic human pathogens found in a variety of habitats, ranging from soil, water, and man-made systems to aerosols (Hilborn et al., 2006; Parker et al., 1983; Vaerewijck et al., 2005). They are well-known colonizers of water distribution systems, and due to their unique cell wall composition may be involved in surface colonization and biofilm formation.

Six *Pseudomonas* OTUs were detected. *P. stutzeri* was the most abundant OTU in CT-2 (28.4%) and was also found in the other two cooling towers. *P. toyotomiensis* (15.7%) represented the second most abundant OTU in CT-2, and was among the persistent shared OTUs (Table S3, Fig. 5). Additional *Pseudomonas* species were seen, but at much lower frequencies. Not considered to be strict pathogens, members of this genus are ubiquitous and metabolically versatile; in addition to their prominent role in biofilm formation some are known as opportunistic human pathogens and cause various infections in e.g. the urinary tract, soft tissues, or the respiratory tract (Goetz et al., 1983; Keys et al., 1983; Potvliege et al., 1987).

Taken together, we couldn't find any evidence for the presence of *sensu stricto* human pathogens in the cooling tower microbial communities analysed in this study, not even among the rare biosphere.

### 3.5 A highly dynamic protist community

We detected the presence of four groups of protists, the Rhizaria, the Excavata, the Alveolata, and the Amoebozoa at all three sampling sites. Although the top taxa in all cooling towers belonged mainly to the Alveolata (Ciliophora and Apicomplexa) and Amoebozoa (Lobosa), we observed pronounced differences in relative abundances between the cooling towers (Fig. 3, Table S4). The most abundant protist groups in cooling tower CT-1 were classified into diverse taxa in the Ciliophora, like *Ancistrum* (9.3%) and *Vorticella* (7.1%), and Lobosa taxa including *Vanella* (8.1%), *Cochliopodium* (7.8%), and *Vexillifera* species (7.7%). In contrast, cooling towers CT-2 and CT-3 were dominated by diverse gregarines. These apicomplexan parasites are often associated with small invertebrates. Although the source of the gregarines in our cooling tower water system remains unknown, they were likely introduced together with their potential animal hosts. Still, there were also several well-known free-living amoebae among the most abundant taxa, such as *Vexillifera*, *Stenamoeba*, *Vannella*, *Hartmannella*, *Echinamoebida*, *Cochliopodium*, *Acanthamoeba*, and *Allovahlkampfia* (Fig. 3, Table S4). Taken together, the protist community detected in the three cooling towers was dominated by taxa frequently found in diverse freshwater habitats and able to persist in an environment characterized by steady water movement.

The protist community composition was more variable than the bacterial microbiome. No persistent protist taxa were observed, and the most frequently recurring OTUs were detected in less than 50% of the samples (Table S4). These included *Stenamoeba, Ancistrum,* and *Vexillifera* (CT-1) and gregarines (CT-2). The repeated occurrence of gregarines (often associated with small invertebrates) suggests that in addition to bacteria-protists interactions, small invertebrates (though not analysed in this study) may also affect the structure of cooling tower microbiomes.

**Fig. 7:**
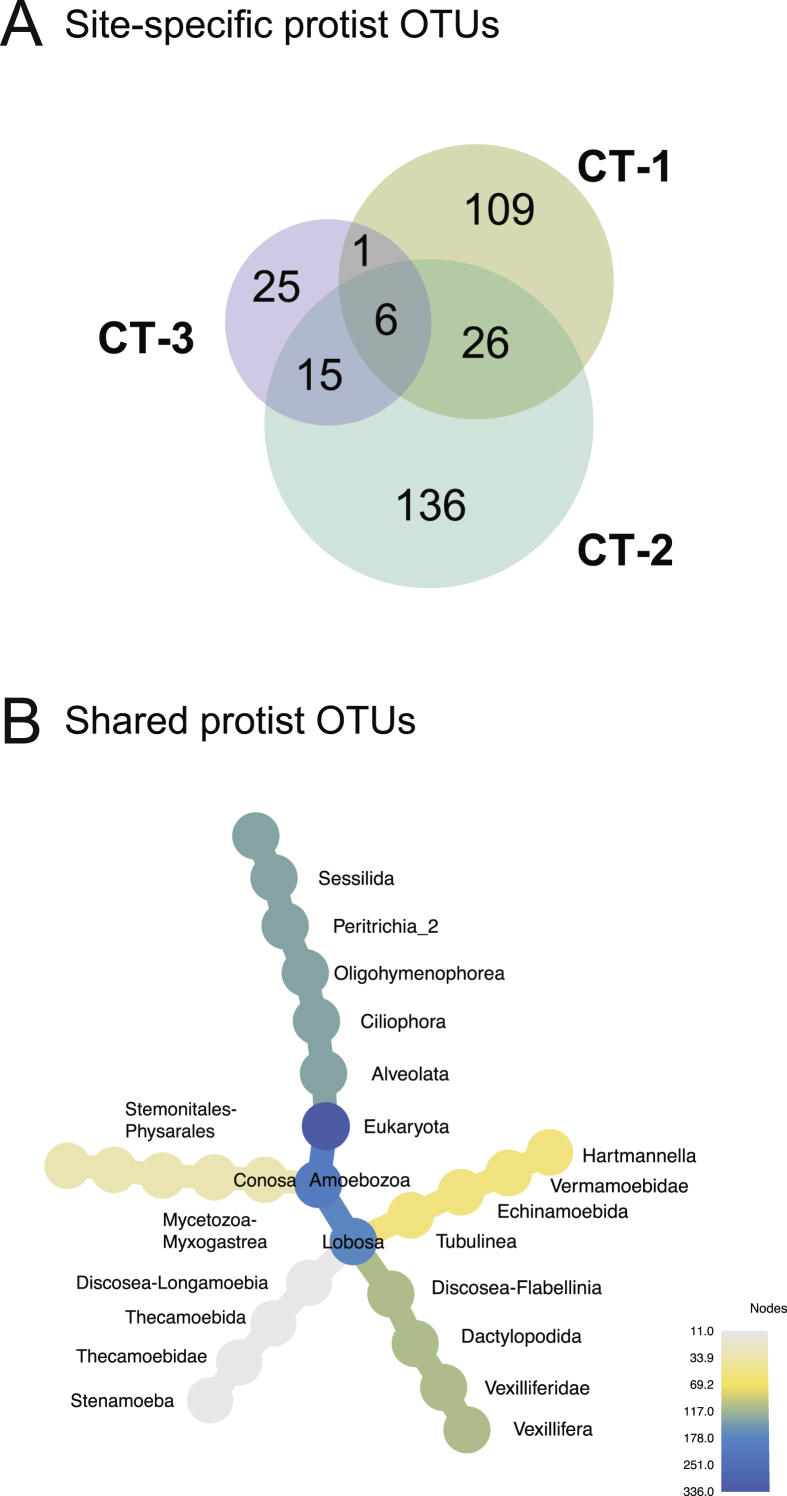
**Site-specific and shared protist OTUs in three cooling towers.** The Venn diagram in **(A)** represents the proportion of shared and site-specific protist OTUs at the three sampling sites. In contrast to the less variable bacterial community composition, the majority of the protist OTUs are site-specific, and the number of OTUs shared by all three sampling sites constitutes only a very small fraction of all OTUs. **(B)** The heat tree indicates the taxonomic classification of the six shared OTUs, most of which are free-living amoebae (*Hartmannella*, *Vexillifera*, *Stenamoeba*). Unclassified taxa are unlabeled.

By far, the largest proportion of taxa was site-specific (Fig. 7A, Table S4). This included representatives of the genera *Stenamoeba*, *Vannella*, *Echinamoeba*, and *Cochliopodium* (Amoebozoa), *Tetrahymena*, *Chilodonella,* and gregarines (Alveolata), *Neobodo* and Vahlkampfiidae (Excavata), and *Paracercomonas* (Rhizaria). More than half of these site-specific OTUs do not occur at more than one time-point, further indicating that the protist community in the water basin is more variable than the bacterial community. Possible reasons for this may include (i) varying protist community composition of the source water, (ii) competition between protists, or (iii) random shearing of protists associated with bacterial biofilms.

There were few OTUs found at all three cooling towers (6 corresponding to 1.7% of all protist OTUs). These included a member of the Sessilida, and free-living amoebae such as *Hartmannella*, *Stenamoeba*, *Vexillifera*, and a conosa member. (Fig. 7B). Taken together, the protist microbiome of cooling tower water is surprisingly diverse, with a species richness lower than the bacterial community but in the same order of magnitude. At the same time, it is highly dynamic and only showed a small core set of persistent and shared taxa.

### 3.6 Bacteria-protist interactions uncovered by co-occurrence networks

**Fig. 8:**
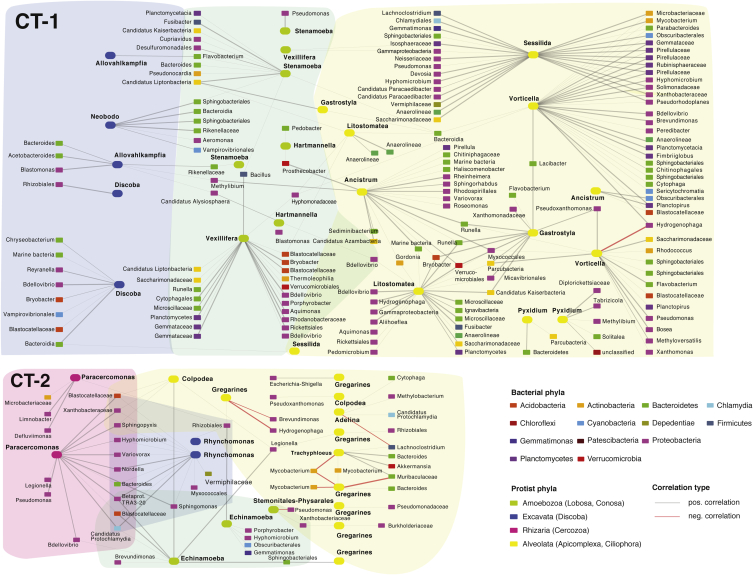
**Co-occurrence network indicating bacteria-protist relationships in cooling tower water microbiomes.** Co-occurrence networks including only interactions between bacterial and protist OTUs are shown for each sampling location; positive correlations are indicated by grey connections (edges), negative correlations are indicated by red edges. The number of negative correlations might be underestimated due to the conservative thresholds applied in this study. The width of the edges negatively correlates to the p-value of each predicted interaction. In CT-1 the majority of the protist nodes are members of the Alveolata, whereas all four protist group are represented in the CT-2 network. This analysis revealed interactions involving known protist-associated bacteria, constituting 15.6% (CT-1) and 24% (CT-2) of all predicted relationships. (For interpretation of the references to color in this figure legend, the reader is referred to the Web version of this article.)

To gain insights into putative interactions between bacteria and protists in the cooling tower water microbiomes, we constructed and analysed co-occurrence networks for each cooling tower using all bacterial and protist OTUs, and a comprehensive approach for sequence abundance-based network inference (Faust and Raes, 2016). In our analysis, we only considered relationships between bacteria and protists (Fig. 8). The resulting co-occurrence networks were characterized by a high number of nodes (189 OTUs for CT-1, 61 for CT-2) and correlations (207 edges for CT-1, 78 for CT-2). Due to the low sample number, CT-3 was not included in this analysis. The networks are composed of mainly positive correlations, in which the abundances of bacterial and protist OTUs followed the same trend.

Protists in the CT-1 network are mainly ciliates (Alveolata; Oligohymenophorea), followed by naked amoebae (Amoebozoa; Discosea). Most protists were correlated with a number of bacterial taxa, with *Vorticella* and an unclassified Sessilida showing highest centrality (i.e. number of edges) identifying them as key players (Fig. 8). Each protist node had its own individual set of bacterial interaction partners. Some bacterial nodes were shared between different protists but the majority (73%) of bacterial nodes were linked to only one protist, suggesting a certain degree of specificity of bacteria-protist interactions. In comparison, the CT-2 network had fewer protist nodes with high centrality. However, we could detect more bacterial taxa, who co-occurred with more than one protist (40%). The most abundant bacterial OTUs included in both networks were members of the Proteobacteria, the Planctomycetes, and the Bacteroidetes.

Remarkably, 15.6% of all predicted bacteria-protist relationships in the CT-1 network and 24% in the CT-2 network were comprised of bacterial genera with members known to associate with protists (Fig. 8). This included several well-known bacterial symbionts of free-living amoebae or ciliates, such as members of the genera *Protochlamydia* (Collingro et al., 2005; Fokin, 2004; Horn, 2008; Vannini et al., 2004) and the family Rickettsiaceae (Horn et al., 1999). Bacterial symbionts of protists are ubiquitous in ciliates and amoeba and in some cases represent ancient relationships originating millions of years ago (Görtz, 2001; Horn, 2008; Molmeret et al., 2005). These bacteria generally show reduced metabolic capabilities and are thus dependent on their protist hosts (Klein et al., 2012; McCutcheon and Moran, 2012; Schmitz-Esser et al., 2010). Some act as defensive symbionts or confer resistance to environmental stress (Dziallas et al., 2012; Fujishima et al., 2005; Grosser et al., 2018; Hori and Fujishima, 2003), but the relevance for the protist host is often unclear.

The networks also included several bacterial groups transiently thriving in protists, such as members of the genera *Legionella, Mycobacterium*, *Pseudomonas, Nordella, Variovorax, Bosea, Acidovorax, Cupriavidus, Brevundimonas,* and *Flavobacterium* (Corsaro et al., 2010; Drancourt, 2014; Evstigneeva et al., 2009; Fields et al., 2002; Greub and Raoult, 2004; La Scola et al., 2004; Michel et al., 1995; Thomas et al., 2008; Zeybek and Binay, 2014). Interestingly, our co-occurrence analysis also predicted members of the groups Parcubacteria (OD1; Corsaro et al., 2010) and Depedentiae (TM6) as interaction partner of protists. This observation further supports a symbiotic lifestyle for Parcubacteria as suggested by (meta-)genomic analysis (Gong et al., 2014; Nelson and Stegen, 2015) and provides additional support for the endosymbiotic lifestyle of Depedentiae members (Delafont et al., 2015). Our analysis underscores the potential of protists in cooling tower water microbiomes as reservoirs for intracellular bacteria, including relatives of opportunistic pathogens (Greub and Raoult, 2004).

Of note, the protist-associated bacteria in our network generally show interactions with several different protist OTUs. This indicates that in a natural setting, bacterial symbionts are more promiscuous and thrive in a wide range of different protists. Among the taxa included in the networks, there are several protists recognized previously as hosts for intracellular bacteria: The amoeba *Vexillifera* is a known host of the environmental chlamydia ‘*Candidatus* Neptunochlamydia vexilliferae’ (Pizzetti et al., 2016). Hartmannella (Vermamoeba) is the natural host of an intranuclear symbiont, ‘*Candidatus* Nucleicultrix amoebiphila‘ and the environmental chlamydia *Rubidus massiliensis* (Bou Khalil et al., 2016; Horn, 2008; Schulz et al., 2014). *Echinamoeba* is a known host for *Pseudomonas aeruginosa* (Michel et al., 1995). In addition, *Allovahlkampfia* which have been suggested to act as hosts for pathogenic bacteria (Mohamed and Huseein, 2016), were also represented in our networks. In this respect, we have recently isolated from the water sample of the tower CT-1 the first testate amoeba, *Cochliopodium minus,* containing a bacterial symbiont (Tsao et al., 2017). Taken together, our co-presence network analysis recovered a large number of known bacteria-protist interactions and suggested that such relationships are much more diverse than recognized currently.

## 4 Conclusions

● Bacterial and protist communities in cooling towers are broadly similar at the class level to those in natural freshwater and drinking water systems. Although community structure can be highly dynamic, the presence of core taxa suggests that cooling tower basins select for biofilm-forming and biofilm-associated microbes.
● Amplicon sequencing techniques that target ribosomal RNA genes is a useful tool to study microbial community dynamics in cooling tower water basins. Longer-term longitudinal studies including outbreak situations are required to better understand the role of the microbial community for propagation and spread of opportunistic bacterial pathogens that have been associated with disease outbreaks. The implementation of upcoming real-time sequencing technologies might facilitate online monitoring of cooling tower communities to predict biofilm formation and colonization with opportunistic pathogens.
● Co-occurrence network analysis is a helpful approach to predict bacteria-protists interactions using amplicon sequencing data. The high number of nodes and degree of connectedness in the networks obtained here highlights the importance of inter-kingdom relationships in cooling tower water systems.
● A design of cooling tower water basins and other measures that prevent biofilm formation should help to decrease the public health risk of cooling towers as a source for bacterial pathogens.

## Author contributions

HFT, US, AI, JW, and MH conceived the study. HFT and US performed the sampling and DNA isolation; HFT performed PCR and prepared sequencing reactions. CH performed the demultiplexing, filtering and clustering of the reads. HFT performed the analysis of the data and prepared the figures; HFT and MH wrote the manuscript. All authors reviewed and edited the manuscript.

## Declaration of interests

All authors declare that there is no conflict of interest.

## Appendix A Supplementary data

Multimedia component 1 **Supplementary Table S1**: Bacterial OTUs removed during the filtering process. Multimedia component 1

Multimedia component 2 **Supplementary Table S2**: Protist OTUs removed during the filtering process. Multimedia component 2

Multimedia component 3 **Supplementary Table S3**: Bacterial OTU table with sampling site specific relative abundance, occurrence, and the taxonomic classification. Multimedia component 3

Multimedia component 4 **Supplementary Table S4**: Protist OTU table with sampling site specific relative abundance, occurrence, and the taxonomic classification. Multimedia component 4

Multimedia component 5 **Supplementary Table S5**: List of opportunistic pathogens and relatives including best blast hits against NCBI database, Silva Living Tree Project database (Version 1.28), and taxonomic classification. Multimedia component 5
